# Deficiency of AtGFAT1 activity impairs growth, pollen germination and tolerance to tunicamycin in Arabidopsis

**DOI:** 10.1093/jxb/erz055

**Published:** 2019-02-18

**Authors:** Kien Van Vu, Chan Young Jeong, Thuy Thi Nguyen, Trang Thi Huyen Dinh, Hojoung Lee, Suk-Whan Hong

**Affiliations:** 1 Department of Molecular Biotechnology, College of Agriculture and Life Sciences, Bioenergy Research institute, Chonnam National University, Gwangju, Republic of Korea; 2 Department of Molecular Biotechnology, College of Life Sciences and Biotechnology, Korea University, Seoul, Republic of Korea

**Keywords:** Arabidopsis, endoplasmic reticulum (ER) stress, GFAT, hexosamine biosynthesis pathway, UDP-GlcNAc, pollen-dependent transmission defect, pollen germination, protein *N*-glycosylation

## Abstract

The hexosamine biosynthetic pathway (HBP) plays essential roles in growth and development in plants. However, insight into the biological function of glutamine:fructose-6-phosphate amidotransferase 1 (GFAT1), mediating the first regulatory step of the HBP, remains unclear in plants. Here, we report the molecular characterization of Arabidopsis *AtGFAT1* gene. *AtGFAT1* was highly expressed in mature pollen grains, but its expression was not detectable in the rest of the organs. Pollen grains bearing the *gfat1-2* knockout allele displayed defects in a polar deposition of pectin and callose in the pollen cell wall, leading to no genetic transmission of the *gfat1-2* allele through the male gametophyte. *AtGFAT1* overexpression increased glucosamine (GlcN) content and enhanced resistance to tunicamycin (Tm) treatment, while RNAi-mediated suppression reduced GlcN content and resistance to Tm treatment. However, the decrease in Tm resistance by RNAi suppression of *AtGFAT1* was recovered by a GlcN supplement. The exogenous GlcN supplement also rescued *gfat1-2/gaft1-2* mutant plants, which were otherwise not viable. The *gfat1-2/gfat1-2* plants stopped growing at the germination stage on GlcN-free medium, but GlcN supplement allowed wild-type growth of *gfat1-2/gfat1-2* plants. In addition, reactive oxygen species production, cell death and a decrease in protein *N*-glycosylation were observed in *gfat1-2/gaft1-2* mutant plants grown on GlcN-free medium, whereas these aberrant defects were not detectable on GlcN-sufficient medium. Taken together, these results show that the reduction of protein *N*-glycosylation was at least partially responsible for many aberrant phenotypes in growth and development as well as the response to Tm treatment caused by *AtGFAT1* deficiency in Arabidopsis.

## Teaser

Suppression of glucose flux into the hexosamine biosynthetic pathway impairs growth and development through at least a partial decrease in protein *N*-glycosylation in the Arabidopsis endoplasmic reticulum.

## Introduction

Living organisms respond to various developmental and environmental variations using a multitude of coordinated signal networks to maintain optimal growth and proliferation (Hardivillé and Hart, 2014). Recently, the hexosamine biosynthetic pathway (HBP) has emerged as one of the key sensors for cellular nutrition because the production of its final product, UDP-*N*-acetylglucosamine (UDP-GlcNAc), is critically dependent on intermediates from a number of metabolic pathways, including glucose, amino acids, fatty acids, and nucleotide (Buse, 2006; Denzel *et al.*, 2014; Yuzwa and Vocadlo, 2014). The first step of the HBP is mediated by glutamine:fructose-6-phosphate amidotransferase (GFAT), converting fructose-6-phosphate and glutamine into glucosamine-6-phosphate (GlcN-6-P) and glutamate (Buse, 2006). Through four enzymatic steps, the HBP provides UDP-GlcNAc, an essential amino sugar donor for glycosylation of proteins and lipids and for biosynthesis of the chitin that forms the cell wall in fungus (Namboori and Graham, 2008).

There are at least four types of protein glycosylations depending on chemical bonding and structural properties: *N*- and *O*-linked glycosylation, *O*-GlcNAcylation, and glypiation (glycosylphosphatidylinositol (GPI) anchor attachment) (Strasser, 2016). First, *N*-linked glycosylation is the most common glycosylation of proteins in the endoplasmic reticulum (ER), attaching a preassembled oligosaccharide at the amide group of an asparagine residue. The attachment of an *N*-linked glycan is indispensable for proper protein folding and assembly of numerous proteins in the ER (Liu and Li, 2014). Disruptions in the *N*-glycosylation process can lead to an accumulation of unfolded proteins in the ER and result in perturbations in the ER homeostatic balance, also known as ER stress. ER stress triggers an array of cellular responses called the unfolded protein response (UPR) to restore ER homeostasis by enhancing protein folding capacity, stimulating ER-associated protein degradation, and attenuating general protein translation (Bao and Howell, 2017). Second, *O*-linked glycosylation is fundamentally different from *N*-linked glycosylation. This modification occurs in the Golgi apparatus. Unlike *N*-glycosylation in which preassembled glycan is attached, a single sugar moiety is transferred to the hydroxyl group of a serine residue on specific proteins (Saito *et al.*, 2014). Third, *O*-linked GlcNAcylation refers to the addition of a single GlcNAc to serine or threonine residues of various cytoplasmic and nuclear proteins in plant and mammalian cells (Hardivillé and Hart, 2014). This post-translational modification is mediated by *O*-GlcNAc transferase (OGT) and exerts significant influence on the stability of proteins and protein–protein interactions by competing with the phosphorylation of serine and threonine residues that control their biochemical activities. To date, two Arabidopsis genes encoding OGTs have been reported, named *SPINDLY* (*SPY*) and *SECRET AGENT* (*SEC*) (Hartweck *et al.*, 2002). Arabidopsis mutants deficient in either *SPY* or *SEC* displayed no detectable defects, whereas no double mutant seedlings were obtained, indicating that the OGT activity plays an essential role in Arabidopsis embryogenesis. Finally, a GPI anchor is attached to proteins on the luminal side of the ER (Fujita and Kinoshita, 2010). The GPI-anchor transamidase complex cleaves the polypeptide chain and concurrently links the carboxyl terminus to the preassembled GPI anchor core through a phosphoethanolamine bridge. The first step of GPI anchor synthesis is to transfer the GlcNAc to inositol of phosphatidylinositol on the cytoplasmic side of the ER, which is mediated by a GPI–*N*-acetylglucosaminyltransferase (GnT) complex. Mutations in *SETH1* and *SETH2*, corresponding to mammalian subunits of the GPI–GnT complex, impair callose deposition in pollen tube, leading to blocking of pollen germination in Arabidopsis (Lalanne *et al.*, 2004).

Recent work on genes encoding enzymes for the second and last steps of the HBP in plants have shown that the HBP is indispensable for plant growth and development, as in animals. For example, single base and knockout mutations in the *GNA* gene in Arabidopsis, encoding GlcN-6-P *N*-acetyltransferase, resulted in growth retardation and a lethal phenotype, respectively (Nozaki *et al*., 2012; Riegler *et al.*, 2012). The T-DNA insertion in *GNA* in rice reduced the wild-type UDP-GlcNAc content by about 90%, giving rise to abnormal root morphology (Jiang *et al.*, 2005). The Arabidopsis genome contains two genes encoding the enzyme *N*-acetylglucosamine-1-P uridylyltransferase (*GlcNAc1p.UT1* and *GlcNAc1p.UT2*) that mediates the last step of the HBP (Chen *et al.*, 2014). Chen *et al.* (2014) demonstrated that a single *glcna.ut1* or *glcna.ut2* knockout mutation displayed no detectable difference, but the double *glcna.ut1 glcna.ut2* mutation was lethal. Although intriguing roles of the HBP in plants are emerging, much of our current understanding on glutamine:fructose-6-phosphate amidotransferase 1 (GFAT1) comes from work in animal systems. For example, a recent study identified gain-of-function (GOF) mutations in *gfat-1* encoding GFAT in *Caenorhabditis elegans* that extend lifespan (Denzel *et al.*, 2014). *gfat-1* GOF mutation was revealed to enhance endoplasmic-reticulum-associated protein degradation (ERAD) protein expression and autophagic activity through increase in GlcN and UDP-GlcNAc levels. Wang *et al.* (2014) showed that mammalian *GFAT* is a direct target of X-box binding protein 1 (XBP1), a highly conserved transcription factor of UPR. Increase in HBP flux by spliced XBP1 (Xbp1s) overexpression was shown to protect the heart from ischemia–reperfusion (I/R) injury in mice.

Hassid *et al.* (1959) first reported the presence of GFAT activity in higher plants. The GFAT activity from *Phaseolus aureus* was partially purified and characterized (Vessal and Hassid, 1972). Despite the ubiquitous presence of the HBP, its physiological functions remain elusive in plants. Herein, we report the functional characterization of *AtGFAT1*, which is present as a single copy in the Arabidopsis genome. *AtGFAT1* is shown to be highly expressed in mature pollen grains and significantly induced under ER stress condition. We showed that T-DNA insertion into *AtGFAT1* had little effect on pollen maturation, but impaired pollen germination. Interestingly, *gfat1-2/gaft1-2* mutant plants were rescued by a GlcN supplement. In addition, a decrease in protein *N*-glycosylation was observed only in *gfat1-2/gaft1-2* seedlings grown on GlcN-free medium, and not detected on GlcN-sufficient medium. Taken together, these results suggest that aberrant phenotypes due to *AtGFAT1* deficiency resulted at least in part from a reduction in protein *N*-glycosylation in Arabidopsis.

## Materials and methods

### Plant materials and growth conditions

All Arabidopsis lines used in this study are of the Col-0 ecotype. T-DNA insertion lines were obtained from the Arabidopsis Biological Resource Center (www.arabidopsis.org): *gfat1-1* (SALK_092218), *gfat1-2* (SALK_058887), *gfat1-3* (SALK_133173), and *qrt1-4* (SALK_024014). For experimental analyses, sterilized seeds were sown on half-strength Murashige and Skoog (MS) medium containing 0.8% agar, 1% sucrose, 0.05% MES (pH 5.7), with or without different chemicals: tunicamycin, dithiothreitol (DTT), NaCl, and glucosamine as described.

Genomic DNA was extracted from individual plants as described by Klimyuk *et al.* (1993). To determine the genotypes, a pair of gene-specific primers was designed for each of the three different alleles (Supplementary Table S1 at *JXB* online). To confirm T-DNA insertion, PCR amplification was performed using the respective gene-specific primer and the T-DNA left border primer LBb1. The precise positions of the T-DNA insertions were determined by sequencing the PCR products with the T-DNA left border primer LBb1.

### RNA isolation, RT-PCR and quantitative real-time PCR

Total RNA was isolated from roots, leaves, stems, and flowers (Lee *et al.*, 2017). For the RT-PCR reaction, 5 µg of total RNA was reverse-transcribed using a Superscript First-Strand Synthesis system (Invitrogen, Carlsbad, CA, USA), followed by RNase H treatment. A 1 µl aliquot of the reverse transcribed reaction was used as a template for PCR with RED Taq DNA polymerase (Sigma-Aldrich, St Louis, MO, USA). Each cDNA sample was diluted 10 times and used for RT-PCR using specific primer pairs listed in Supplementary Table S1. PCR cycling conditions were as follows: 94 °C for 1 min, followed by 25 cycles at 94 °C for 1 min, 62 °C for 30 s, and 72 °C for 30 s, and a final elongation step at 72 °C for 15 min. Amplified products were visualized on a 1.2% (w/v) agarose gel. *ACTIN2* was used as the internal normalization control.

Quantitative real-time PCR for *AtGFAT1*, *CNX1*, *BIP1/2*, and *BIP3* was performed using Takara SYBR Premix Ex Taq on the Takara Thermal Cycler Dice^TM^ Real Time System (Takara Bio Inc., Kyoto, Japan). Amplification was assessed in real time using the iCycler iQ system Software version 3.0 (Bio-Rad, Hercules, CA, USA). Each reverse transcript was quantified in duplicate, and the results were obtained from three separately prepared RNA samples (Jeong *et al.*, 2018). The thermal cycling conditions were 40 cycles at 95 °C for 10 s for denaturation and 60 °C for 30 s for annealing and extension.

### Histochemical assay, tissue staining, and cell death

Histochemical analysis of β-glucuronidase (GUS) activity was conducted as previously described (Jefferson, 1989). The previously generated transgenic Arabidopsis harboring the *pCNX1::GUS* construct was used as a marker for ER stress in GUS histochemical staining (Vu *et al.*, 2017). GUS-stained tissues shown herein are indicative of the typical result out of three independent transgenic lines. For 4′,6-diamidino-2-phenylindole (DAPI) staining, mature pollen grains were soaked in 0.1 M DAPI staining solution and observed using a fluorescence microscope as previously described by Veley *et al.* (2014). The viability of pollen grains was assessed as described previously (Alexander, 1969). For scanning electron microscopy, pollen quartets from *qrt1-4/qrt1-4* and *qrt1-4 gfat1-2/+* plants were dusted onto the surface of carbon sticky tape using dissecting forceps and then observed using the Hitachi TM-1000 table-top scanning electron microscope (Hitachi High-Technologies Corp.). For staining callose and pectin, pollen grains were incubated without fixation in germination medium containing calcofluor white (0.001%, w/v) and ruthenium red (0.01%, w/v), respectively (Li *et al.*, 2017). *In vitro* germination of the Arabidopsis pollen grains was performed in acid-washed depression slides in a liquid medium, as previously described (Fan *et al.*, 2001). Pollen grains from *qrt1-4/qrt1-4* and *qrt1-4 gfat1-2/+* plants were carefully tapped onto the slides, and the medium was added. Slides were then incubated in a humid chamber at 24 °C for 24 h in the dark. Staining for H_2_O_2_ production and lesion formation was performed on 2-week-old seedlings with 3,3′-diaminobenzidine (DAB) solution and trypan blue staining as described (Veley *et al.*, 2014).

### *Construction of* pAtGFA1::GUS, AtGFAT1 *overexpression, and RNAi vector*

For the *pAtGFA1::GUS* construct, the 1171-bp promoter fragment from −1197 to −26 of the *AtGFAT1* genomic DNA was amplified (the A site of ATG, the translation start codon, was designated as +1). The resulting fragment containing the *AtGFAT1* promoter was inserted into *Bam*HI and *Hin*dIII sites of the pBI121 binary vector. To construct the *AtGFAT1* overexpression construct, the full-length *AtGFAT1* cDNA was amplified using the primer pair GFAT1 cDNA:F–GFAT1 cDNA:R, and cloned into the pGEM-T easy vector (Promega, Madison, WI, USA). Then the full-length *AtGFAT1* cDNA was inserted into *Bam*HI and *Sac*I sites of pBI121. For *AtGFAT1* RNAi construction, a pair of sense and antisense fragments of *AtGFAT1* 601 bp in length were amplified using two primer pairs, GFAT1 RNAi Sense:F–GFAT1 RNAi Sense:R and GFAT1 RNAi Antisense:F–GFAT1 RNAi Antisense:R (listed in Supplementary Table S1). *AtGFAT1* RNAi sense and antisense fragments then were inserted into the *Asc*I/*Swa*I and *Bam*HI/*Xba*I restriction sites at both ends of the CHSA intron of the pFGC5941 vector, respectively. For complementation, *pGFAT1::GFAT1* was constructed by replacing the cauliflower mosaic virus (CaMV) 35S promoter of the *AtGFAT1* overexpression construct with the 1171-bp *AtGFAT1* promoter fragment. All the cloning PCRs were performed by the proofreading Solg^TM^*Pfu* DNA polymerase (SolGent Co., Seoul, Korea). The integrity of PCR products had been confirmed by sequencing before substitution for the corresponding regions in the binary vectors.

The resulting binary vectors were introduced into *Agrobacterium tumefaciens* strain GV3101 (Holsters *et al.*, 1980) for Arabidopsis transformation by the floral dip method (Clough and Bent, 1998). Seedlings from the T1 generation were selected on 1/2 MS medium containing 50 µM phosphinothricin or 50 µg ml^−1^ kanamycin, depending on the binary vectors used for transformation (phosphinothricin for the pFGC5941 vector and kanamycin for the pBI121 vector). T3 progeny homozygous for each transgene were isolated and three independent lines for each construct were selected for further examinations.

### Glucosamine and UDP-GlcN measurement

Glucosamine level was measured using the K-GAMINE D-glucosamine test kit according to the manufacturer’s instructions (Megazyme, Wicklow, Ireland). Twelve-day-old seedlings were frozen in liquid nitrogen, followed by grinding to fine powders and then dissolving in 5 ml deionized water. The samples were centrifuged for 10 min at 3000 *g*, and the supernatant was carefully filtered through a paper filter (Whatman no. 1) to remove any insoluble particles. From each sample, 1 ml was pipetted into the cuvettes for enzymatic chemical reactions. The glucosamine amount was measured through the formation of reduced NADPH by the increase in absorbance at 340 nm, which is directly proportional to the GlcN concentration.

Using an HPLC system (L-4200H UV-VIS detector, 655A-52 column oven, D-2500 chromatographic-data processor; Hitachi), UDP-GlcN was quantitatively measured in 12-day-old seedlings as previously described (Carpita and Delmer, 1981) with a few modifications. The amount of UDP-GlcNAc was estimated by comparison of the peak area detected by absorbance at 254 nm of samples and reference solutions of known concentrations of UDP-GlcNAc.

### Concanavalin binding assay

To detect N-glycan on proteins, total proteins (20 μg) from 2-week-old seedlings of each genotype were resolved by 12% SDS-PAGE gel and transferred onto a nitrocellulose membrane. The membrane was incubated in blocking solution (1% BSA in phosphate-buffered saline (PBS)) for 30 min and then in blocking solution containing 1 mg l^−1^ concanavalin A (Con A)–alkaline phosphatase conjugate (Sigma-Aldrich, St Louis, MO, USA) for 1 h at 25 °C. Membranes then were washed three times with PBS containing 0.05% Tween 20 for 5 min each, rinsed once with alkaline phosphatase buffer (100 mM Tris, pH 9.3, and 100 mM NaCl). Glycoproteins were detected using an enhanced chemiluminescence detection kit (Thermo Fisher Scientific, Waltham, MA, USA) and recorded with the Odyssey Fc Imaging System (LI-COR Biosciences, Lincoln, NE, USA).

## Results

### AtGFAT1 is expressed exclusively in mature pollen grains and strongly induced under tunicamycin stress

**Fig. 1.**
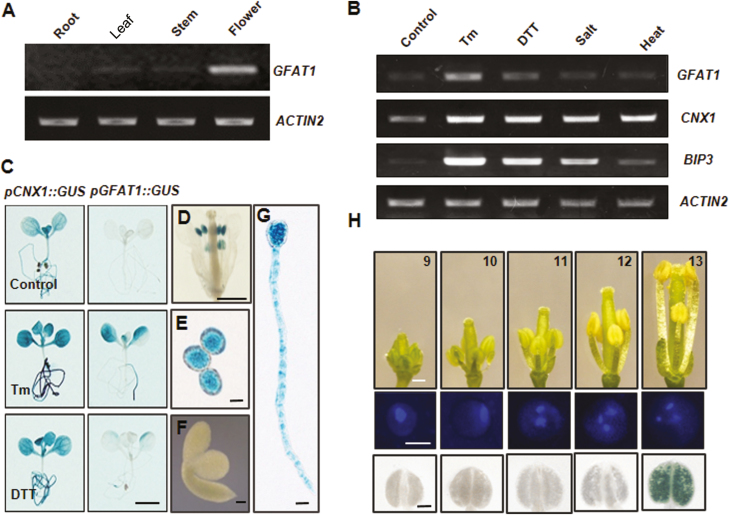
Expression analysis of *AtGFAT1*. (A) RT-PCR analysis of *AtGFAT1* in roots, leaves, stems, and flowers from wild-type plants. (B) RT-PCR analysis of *AtGFAT1* in wild-type plants after various stress treatments. Total RNAs were extracted from 2-week-old seedlings treated for 24 h with 50 ng ml^−1^ Tm, 1 mM DTT, 100 mM NaCl (salt), or 37 °C (heat). *AtCNX1* and *AtBIP3* were used as ER stress controls and *ACTIN2* was used as an internal normalization control. (C) Histochemical analysis for 10-day-old seedlings of transgenic plants harboring *pGFAT1::GUS* or *pCNX1::GUS* construct treated for 24 h at 50 ng ml^−1^ Tm or 1 mM DTT. The same transgenic plants grown under normal conditions were used as a control. (D–G) GUS staining for *pGFAT1::GUS* expression at different developmental stages: mature flower (D), pollen grains (E), embryo of germinating seed (F), and pollen tubes (G). Different stages of flowers from *pGFAT1::GUS* transgenic lines were sampled for GUS staining (H). Flowers were laid out in order of developmental stages as indicated by the numbers (top). Representative pollen grains (middle) and anthers (bottom) are shown. Scale bars: 3 mm (C), 0.5 mm (D), 0.2 mm (F), 10 µm (E, G), 0.5 mm (H, top), 10 µm (H, middle), 1 mm (H, bottom).

The deduced amino acid sequence of AtGFAT1 displays high similarity to those from other organisms including *E coli*, nematode, and mice (Supplementary Fig. S1). However, simple sequence comparison alone was not sufficient to determine the biological functions of *AtGFAT1* in Arabidopsis. To yield insights into the physiological roles of the HBP in plants, we firstly performed RT-PCR analysis to examine the expression profile of *AtGFAT1* mRNA in various organs of Arabidopsis. As shown in Fig. 1A, the mRNA level of *AtGFAT1* was abundantly detected in flowers, whereas its expression was barely observed in roots, leaves, and stems. Recent studies from animal systems revealed that mouse *GFAT1* is up-regulated directly by spliced Xbp1, the active transcription factor in the ER stress signaling pathway (Wang *et al.*, 2014). In plants, ER stress is published as being induced by abiotic stresses, such as high temperature and high salinity as well as two chemicals, tunicamycin (Tm), an inhibitor of *N*-glycosylation in ER that disturbs the protein folding machinery, and DTT, a reducing agent that blocks disulfide-bond formation of cytosolic and ER proteins (Koiwa *et al.*, 2003; Gao *et al.*, 2008; Wang *et al.*, 2011). As shown in Fig. 1B, Tm treatment for 24 h resulted in induction of *AtGFAT1* mRNA, which was comparable to up-regulation of ER stress-responsive marker genes (*AtBiP3* and *AtCNX1*). In response to DTT, its expression was slightly increased and the effect of NaCl and high temperature treatment was hardly detected.

To further investigate the *AtGFAT1* expression patterns, the 1171-bp promoter fragment from −1197 to −26 of the *AtGFAT1* genomic DNA (the A site of ATG, the translation start codon, was designated as +1) was fused with the *GUS* gene. The transgenic lines expressing GUS driven by the *AtGFAT1* promoter (*pGFAT1::GUS*) were generated via *Agrobacterium*-mediated transformation. Using 10-day-old transgenic lines bearing the *pGFAT1::GUS* construct, we also confirmed the significant induction of the *AtGFAT1* promoter activity by Tm, but not by DTT (Fig. 1C). The tissue-specific expression of *AtGFAT1* was also examined by histochemical staining of *pGFAT1::GUS* transgenic lines. GUS staining was strongly detected in mature anthers (Fig. 1D), mature pollen grains (Fig. 1E) and pollen tubes (Fig. 1G), but not in other reproductive organs, including sepals, stamen filaments, and pistil (Fig. 1D). No GUS staining was detected in seedlings at 1 h after seed imbibition (Fig. 1F).

We further dissected its detailed expression pattern during floral development. As shown in Fig. 1H, the *AtGFAT1* promoter was highly active only in the late tricellular pollen grains, corresponding to the mature flower onwards (stage 13; floral developmental stages according to Smyth *et al.*, 1990), but GUS staining was barely detectable in mono-, bi-, and early tricellular pollen grains.

### *Disruption of* AtGFAT1 *results in male gametophytic sterility*

**Fig. 2.**
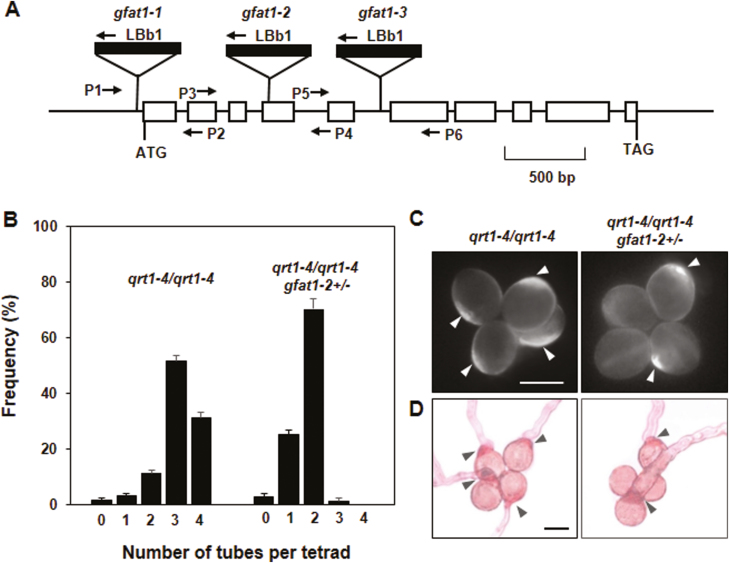
*gfat1-2* pollen is defective in the polar deposition of cell wall materials. (A) Schematic representation of *AtGFAT1* genomic DNA and three sites of T-DNA insertion. Boxes represent exons and lines represent introns. LBb1 and three primer pairs, P1–P2, P3–P4, and P5–P6, were used to identify *gfat1-1*, *gfat1-2*, and *gfat1-3* alleles, respectively. (B) The frequency of pollen quartets from *qrt1-4*/*qrt1-4* and *qrt1-4*/*qrt1-4 gfat1-2/+* mutant plants bearing zero to four pollen tubes after 16 h incubation. Values represent the means ±standard error (SE) from three independent repeats (*n*≥100 per repeat). Pollen quartets from *qrt1-4*/*qrt1-4* and *qrt1-4*/*qrt1-4 gfat1-2/+* plants were stained with calcofluor white (C) or with ruthenium red (D). Scale bars: 20 µm (C, D). Pollen grains showing polar deposition of callose and pectin are indicated with arrowheads.

To facilitate the functional analysis of *AtGFAT1* (At3g24090), we analysed three T-DNA insertion lines from the Arabidopsis Biological Resource Center. As confirmed by PCR and sequencing analysis, one of these lines, named *gfat1-1*, contained a T-DNA insertion 24 bp upstream of the initiation codon, whereas in the other two alleles, *gfat1-2* and *gfat1-3*, T-DNA was inserted in the fourth exon and the fifth intron, respectively (Fig. 2A). The progenies of self-pollinated heterozygous plants were genotyped by PCR using gene- and T-DNA-specific primers to identify homozygous mutant lines for each T-DNA insertion (Table 1). Among 153 progenies from self-pollinated *gfat1-1*/+ plants, we detected 37 wild-type, 77 *gfat1-1*/+, and 39 *gfat1-1/gfat1-1* plants. These numbers are very close to those expected by Mendelian segregation. However, the self-fertilization of heterozygous *gfat1-2*/+ and *gfat1-3*/+ plants produced offspring in the segregation ratios of 275:268:0 and 257:276:0 for wild-type, heterozygous, and homozygous plants, respectively (Table 1). These genetic results reveal a typical 1:1 segregation distortion, suggesting a defect in genetic transmission through either the male or the female gametophyte. To determine the precise cause of this transmission deficiency, we performed reciprocal cross-pollinations of *gfat1-2/+* and *gfat1-3/+* plants to wild-type plants, and counted the number of each genotype of their progenies (Table 1). When both the *gfat1-2/+* and *gfat1-3/+* plants were used as pollen donors, none of the resulting progenies carried either of the two T-DNA insertion alleles. However, the crosses using T-DNA insertion lines as maternal parents allowed recovery of *gfat1-2* and *gfat1-3* alleles (52.8% and 51.4%, respectively). Based on these results, we concluded that male-dependent transmission of both *gfat1-2* and *gfat1-3* alleles was completely defective.

**Table 1.** Genetic transmission analysis of *gfat1* mutations

Self-crosses				
Parental genotype	Wild-type progeny^a^	Heterozygous progeny^a^	Homozygous progeny^a^	*P* ^b^
*gfat1-1*/+	37	77	39	0.97
*gfat1-2*/+	275	268	0	<0.00001
*gfat1-3*/+	257	276	0	<0.00001
Reciprocal crosses				
Maternal parent	Paternal parent	+/+ progeny^a^	*gfat*/+ progeny^a^	*P* ^b^
Col-0	*gfat1-2*/+	89	0	<0.00001
*gfat1-2*/+	Col-0	42	47	0.60
Col-0	*gfat1-3*/+	75	0	<0.00001
*gfat1-3*/+	Col-0	67	71	0.73

^a^The progeny genotype was scored by PCR reaction with different primer combinations, as described in ‘Materials and methods’.

^b^ *P* values for 1:2:1 and 1:1 for self-crosses and reciprocal crosses, respectively; validation by χ^2^ test.

### *Polar deposition of cell wall materials is abolished in pollen grains bearing the* gfat1-2 *allele*

For further analyses of defects in pollen-mediated transmission, *gfat1-2/+* was introgressed into the *quartet1-4* (*qrt1-4*) mutant background where four fully functional pollen grains remain attached to form quartets by preventing separation of microsporocyte meiosis products (Preuss *et al.*, 1994). Therefore, half of the pollen quartet produced by *qrt1-4*/*qrt1-4 gfat1-2/+* are mutant pollen grains (*gfat1-2*) and the other two are wild-type pollen grains (*AtGFAT1*). Morphological comparison by scanning electron microscopy revealed no difference between the quartets from *qrt1-4/qrt1-4* and *qrt1-4*/*qrt1-4 gfat1-2/+* plants (Supplementary Fig. S2A). Using DAPI and Alexander staining analysis, we also confirmed no detectable difference in nuclear division or viability of quartets from *qrt1-4*/*qrt1-4 gfat1-2/+* plants (Supplementary Fig. S2B, C). To further address the effect of *gfa1-2* mutation on pollen germination, we performed *in vitro* germination of pollen grains from the *qrt1-4*/*qrt1-4* mutant and *qrt1-4*/*qrt1-4 gfat1-2/+* mutant plants. As shown in Fig. 2B, approximately 86.3% of quartets (*n*=364) from *qrt1-4/qrt1-4* plants displayed three to four pollen tubes, but only 4.2% of those (*n*=319) from *qrt1-4*/*qrt1-4 gfat1-2/+* plants had three pollen tubes, and no quartets with four pollen tubes were found. To corroborate the function of *AtGFAT1* on pollen germination, we examined cell wall deposition of at least 50 quartets from each plant using calcoflour white and ruthenium red staining, which can specifically stain callose and pectin, respectively (Li *et al.*, 2017). In contrast to polar deposition of callose and pectin in all four pollen grains of quartets from *qrt1-4*/*qrt1-4* plants, only two pollen grains displayed polar deposition in quartets from *qrt1-4*/*qrt1-4 gfat1-2/+* plants (Fig. 2C, D). Moreover, pollen tubes were not found in pollen grains with no polar deposition in quartets from *qrt1-4*/*qrt1-4 gfat1-2/+* plants, supporting that the *gfat1-2* mutation affects male-dependent genetic transmission through disrupting polar deposition in pollen grains.

To further confirm that male gametophytic sterility was caused by the loss of GFAT1 activity, we introduced the *AtGFAT1* cDNA under its own promoter into *gfat1-2/+* plants. Transgenic plants homozygous for *pAtGFAT1*::*AtGFAT1* in the *gfat1-2/+* background were isolated to confirm genetic segregation of the *gfat1-2* allele. We were able to identify the *gfat1-2* homozygous plants only under the presence of *pAtGFAT1*::*AtGFAT1* transgene. The complementation plants (*gfat1-2/gfat1-2* plants bearing *pAtGFAT1*::*AtGFAT1*) were morphologically indistinguishable from the wild-type plant under normal conditions (Supplementary Fig. S3A, B). Moreover, *in vitro* pollen germination assays showed normal pollen germination and pollen tube growth in the complemented plants (Supplementary Fig. S3C).

### The level of AtGFAT1 transcripts determine the HBP flux in Arabidopsis

**Fig. 3.**
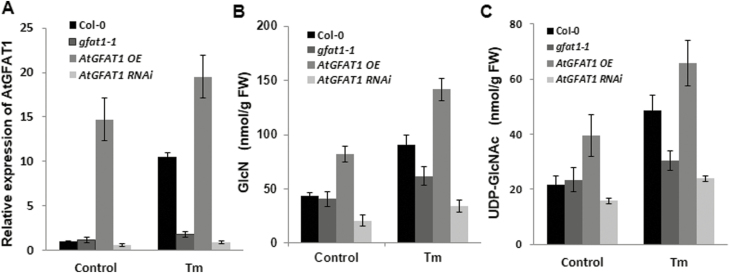
Expression of *AtGFAT1* affects the HBP flux in Arabidopsis. (A) Quantitative RT-PCR analysis for *AtGFAT1* transcript levels. Total RNA was extracted from 12-day-old seedlings of the indicated genotype grown under normal conditions or treated for 6 h with 50 ng ml^−1^ Tm. The *AtGFAT1* expression levels were normalized to transcript levels for the reference gene *ACTIN2*, and then to *AtGFAT1* transcript level in wild-type plants grown under normal conditions, which was set to 1. Values represent mean ±SE from three independent repeats. GlcN (B) and UDP-GlcNAc (C) contents in 12-day-old seedlings of the indicated genotype grown under normal conditions or treated for 6 h with 50 ng ml^−1^ Tm (fresh weight). In particular, for *AtGFAT1* OE and RNAi lines, qRT-PCR analysis and measurement of HBP intermediates were conducted each time on all three independent transgenic lines. Values represent means ±SE of three independent repeats.

To investigate the effect of *AtGFAT1* expression on the HBP flux in Arabidopsis, *AtGFAT1* overexpression (OE) and RNAi lines were generated (Supplementary Fig. S4). Quantitative real-time RT-PCR (qRT-PCR) was performed to examine the *AtGFAT1* transcript levels in 12-day-old seedlings of these transgenic plants and *gfat1-1* mutant plants with T-DNA in its promoter (Fig. 3A). Under the normal conditions, the *AtGFAT1* transcripts increased by approximately 25% in *gfat1-1* and by 13-fold in *AtGFAT1* OE lines compared with that in wild-type plants, while it was reduced by 30–50% in RNAi lines. Tm treatment caused about 10 times and 30% increase in *AtGFAT1* transcripts in wild-type and *AtGFAT1* OE lines, respectively, compared with those in the wild and *AtGFAT1* OE lines under the normal conditions. Although Tm treatment led to about a 50% increase in *AtGFAT1* transcripts in *gfat1-1* and *AtGFAT1* RNAi lines, their expression levels were significantly lower than that of wild-type plants treated equally with Tm.

The Tm-mediated induction of *AtGFAT1* led to the identification of the ER stress-responsive element (ERSE) in its promoter, which is involved in the expression of ER-stress responsive genes. The *cis*-acting element called ERSE (consensus sequence CCAAT-N9-CCACG) has been well documented to have the bipartite regulatory motif in mammals and higher plants (Iwata and Koizumi, 2012). We were able to identify the canonical ERSE motif (CCAAT-N9-CCACG) at 173 bp upstream of the translation initiation site of the *AtGFAT1* promoter (Supplementary Fig S5). As in mammals showing strong conservation of spacing (9 bp) between two elements, the CCAAT element is located 9 bp upstream of the CCACG element in the *AtGFAT1* promoter. As shown in Fig. 3A, the significantly low levels of *AtGFAT1* transcripts following Tm treatment in *gfat1-1* with T-DNA insertion downstream from ERSE supports a role of ERSE in the expression of *AtGFAT1* in response to Tm.

GFAT mediates the conversion of fructose-6-phosphate to GlcN-6-phosphate. Therefore, we speculated that genetic manipulation in *AtGFAT1* transcripts levels may affect the synthesis of GlcN and UDP-GlcNAc. To examine this possibility, we measured the content of GlcN and UDP-GlcNAc in 12-day-old seedlings of *gfat1-1*, *AtGFAT1* OE, and RNAi plants. Consistent with decrease in *AtGFAT1* expression, *AtGFAT1* RNAi plants were revealed to have about 50% and 70% of wild-type levels of GlcN and UDP-GlcNAc, respectively (Fig. 3B, C). Interestingly, *AtGFAT1* OE plants with 13-fold increase in its mRNA showed only twice as much GlcN and UDP-GlcNAc as wild-type plants. These results showed that the levels of *AtGFAT1* transcripts are not directly proportional to the content of GlcN and UDP-GlcNAc, but generally, higher *AtGFAT1* transcripts lead to an increase in their contents in Arabidopsis.

### GlcN levels determine the resistance to ER stress caused by tunicamycin treatment

**Fig. 4.**
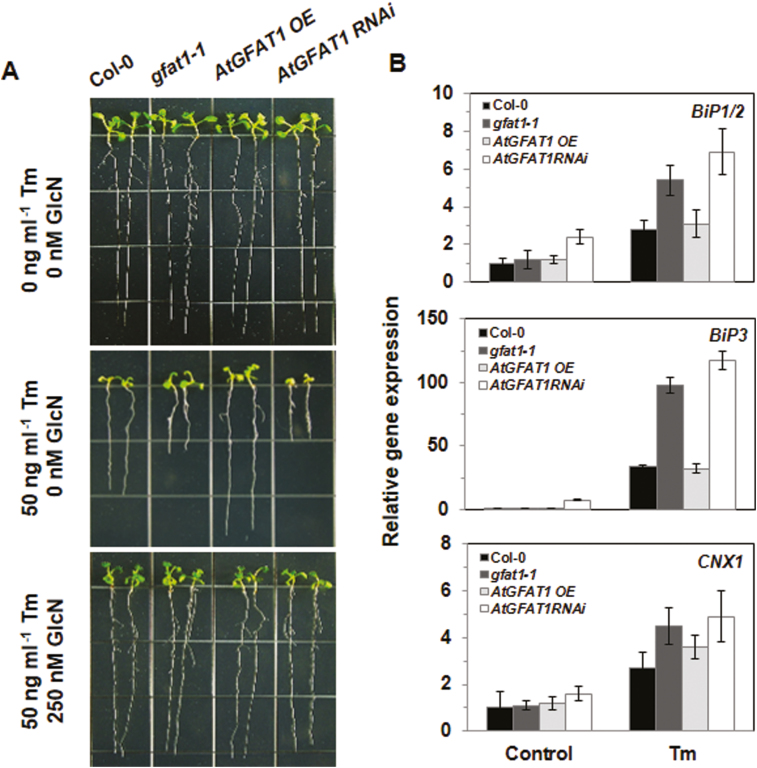
Tunicamycin-mediated inhibition of root growth. (A) Representative 10-day-old seedlings with the indicated genotypes grown on 1/2 MS medium supplemented with Tm and GlcN at the indicated conscentrations. (B) Quantitative RT-PCR analysis for *BIP1/2*, *BIP3*, and *CNX1* transcript levels. Their expression levels were normalized to transcript levels for the reference gene *ACTIN2*, and then to their transcript level in wild-type plants grown under normal conditions, which were set to 1. Values are normalized to *ACTIN2* expression levels and represent means ±SE of three repeats.

Gene expression is generally perceived to be regulated by the requirement of cells or tissues (Liao and Weng, 2015). To examine the effect of alteration in GlcN levels on Tm resistance, we investigated the response of *gfat1-1* and transgenic plants to Tm treatment. When grown on medium containing 50 ng ml^−1^ Tm, primary root growth was inhibited by approximately 53% and 48% in *gfat1-1* and *AtGFAT1* RNAi lines, respectively, and its overexpression resulted in increased root growth by 43% as compared with that of wild-type plants (Fig. 4A). The root growth of plants of each genotype grown on media containing Tm was consistent with the GlcN and UDP-GlcNAc contents of them: *AtGFAT1* OE lines with higher contents of GlcN and UDP-GlcNAc showed a faster rate of root growth compared with that of wild-type plants, while *gfat1-1* and *AtGFAT1* RNAi lines having lower contents of GlcN and UDP-GlcNAc displayed shorter roots than wild-type plants when grown on Tm-containing medium. Moreover, the exogenous GlcN supplement restored the Tm-mediated inhibition of root growth of *gfat1-1* and *AtGFAT1* RNAi lines to that of *AtGFAT1* OE lines. However, DTT treatment had little effect on root growth of *gfat1-1*, *AtGFAT1* OE, and RNAi plants relative to its effect on wild-type plants (Supplementary Fig. S6). These findings are consistent with there being no induction of GUS activity by DTT in *pGFAT1::GUS* transgenic lines (Fig. 1C).

To investigate the effect of altered *AtGFAT1* transcript levels on the expression of ER chaperone genes, qRT-PCR analysis was performed to determine transcript levels of ER stress-responsive genes (Fig. 4B). Total RNA was extracted from 2-week-old seedlings of wild-type, *gfat1-1*, *AtGFAT1* OE, and RNAi plants grown under normal conditions or treated with 50 ng ml^−1^ Tm for 24 h. In the normal condition, a significant increase in *BiP1/2* and *BiP3* transcripts were displayed only in *AtGFAT1* RNAi plants. Tm treatment for 24 h significantly increased *BiP1/2*, *BiP3*, and *CNX1* transcripts in *gfat1-1* and *AtGFAT1* RNAi plants compared with wild-type plants, whereas there was no significant change in the expression levels in *AtGFAT1* OE plants under either the unstressed or ER-stressed condition. These results suggest that the decrease in *AtGFAT1* transcripts in ER stress conditions requires more chaperones involved in protein folding in the ER.

### The reduction of *AtGFAT1* transcript levels leads to growth retardation and a decrease in seed yield under normal conditions

**Fig. 5.**
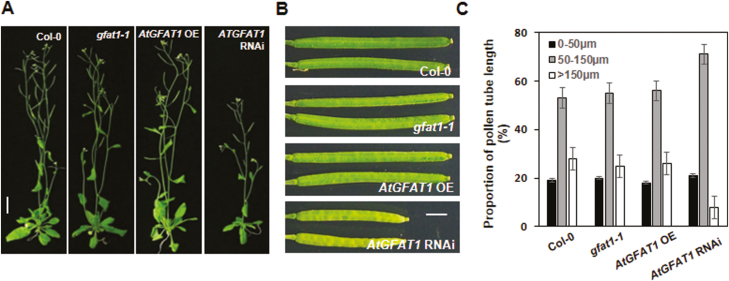
The decrease in *AtGFAT1* transcripts impairs vegetative and reproductive development in Arabidopsis. (A) Photographs of representative 6-week-old plants with the indicated genotypes grown under normal conditions. (B) Mature siliques in plants with the indicated genotypes. (C) Length distributions of pollen tube from plants with the indicated genotypes. Black, gray, and white boxes indicate relative proportion of pollen grains with pollen tube of less than 50 µm, 50–100 µm and longer than 150 µm, respectively. Values represent means ±SE of three repeats (pollen numbers, *n*≥100 per repeat). Scale bar: 1.5 cm (A), 0.2 cm (B).

To examine the physiological roles of GlcN and UDP-GlcNAc in Arabidopsis, we compared the growth of *gfat1-1*, *AtGFAT1* OE, and RNAi lines with wild-type plants under normal conditions. As shown in Fig. 5A, *gfat1-1* and *AtGFAT1* OE plants were wild-type in appearance, whereas *AtGFAT1* RNAi lines displayed an approximate 60–70% reduction of inflorescence height in 6-week-old plants compared with that of wild-type plants. The high expression of *AtGFAT1* in mature pollen grains and no germination of pollen grains containing *gfat1-2* allele led us to investigate the effect of altered levels of *AtGFAT1* transcripts on seed yield and pollen germination in plants. As shown in Fig. 5B and Supplementary Table S2, silique length and seed yield of *gfat1-1* and *AtGFAT1* OE plants were indistinguishable from those of wild-type plants, while those of *AtGFAT1* RNAi plants decreased by about 60% and 75%, respectively, compared with wild-type plants. We also compared pollen germination and pollen tube length of *gfat1-1* and transgenic plants with wild-type plants. When at least 300 pollen grains were cultured *in vitro* at 22 °C for 6 h, there was about 85% pollen germination rates in *gfat1-1* and transgenic plants, which was very similar to that of wild-type plants (data not shown). However, pollen tubes carrying the *AtGFAT1* RNAi construct were shorter than those of wild-type plants. After incubation for 6 h, about 30% of pollen tubes in wild-type, *gfat1-1*, and *AtGFAT1* OE lines were longer than 150 μm, but less than 10% in *AtGFAT1* RNAi lines (Fig. 5C).

### *The lethal phenotype of homozygous* gfat1-2 *mutant plants is averted by exogenous supplementation of GlcN*

The recovery of the wild-type level of Tm resistance in *gfat1-1* and *AtGFAT1* RNAi lines by exogenous GlcN treatment led us to investigate whether it can rescue the lethal phenotype of *gfat1-2* mutant plants. We sprayed 1 or 10 mM GlcN solution once a day on the leaves of 4-week-old *qrt1-4*/*qrt1-4 gfat1-2*/+ plants until we harvested the seeds, and then performed PCR to determine the genotypes of their progenies. The *gfat1-2/gfat1-2* plants were successfully identified among all descendants of *gfat1-2*/+ plants sprayed with both 1 mM and 10 mM GlcN solution, even though their number varied significantly. These results strongly suggest that the exogenous GlcN supplement enabled successful germination of pollen grains bearing the *gfat1-2* allele.

**Fig. 6.**
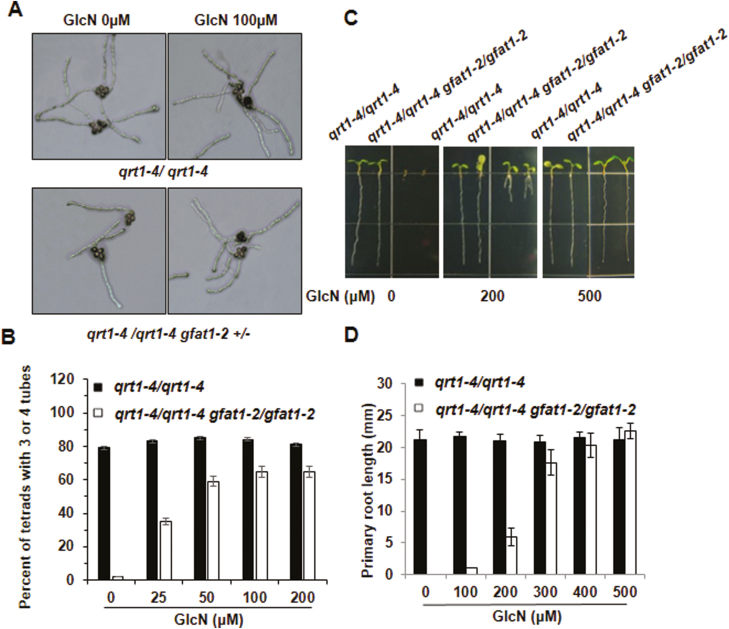
Exogenous GlcN treatment rescues the homozygous *gfa1-2* plants. (A) *In vitro* germination of pollen quartets. Photographs of representative quartets from indicated genotypes were taken after 6 h incubation at 22 °C. (B) The frequency of pollen quartets bearing three to four pollen tubes with the indicated genotypes after 6 h incubation. Values represent means ±SE of three repeats (pollen numbers, *n*≥120 per repeat). (C) Photographs of 10-day-old representative plants with the indicated genotypes grown on 1/2 MS medium containing various GlcN concentrations. Scale bar: 2.0 cm. (D) Histogram showing the primary root length of plants with the indicated genotypes grown for 10 d on 1/2 MS media containing the indicated GlcN concentrations. The mean root length was determined from three independent repeat (*n*=25 per repeat).

To test whether GlcN deficiency is the major defect in germination of pollen grains harboring the *gfat1-2* allele, *in vitro* pollen germination was performed with quartets from *qrt1-4*/*qrt1-4* and *qrt1-4*/*qrt1-4 gfat1-2*/+ plants on media containing GlcN. As shown in Fig. 6A, B, more than 80% of quartets (*n*≥360) from *qrt1-4*/*qrt1-4* plants displayed three or four pollen tubes at all GlcN concentrations, whereas less than 3% of the *qrt1-4*/*qrt1-4 gfat1-2*/+ quartets (*n*≥360) had three or four pollen tubes on GlcN-free media. However, the portion of *qrt1-4*/*qrt1-4 gfat1-2*/+ quartets having three or four pollen tubes increased along with exogenous GlcN amounts, reaching the highest value (about 60%) at 100 μM GlcN.

To further understand the effect of GlcN deficiency on growth, we measured the primary root growth of *qrt1-4*/*qrt1-4* and *qrt1-4*/*qrt1-4 gfat1-2/gfat1-2* plants grown for 5 d on 1/2 MS media containing various GlcN concentrations (Fig. 6C, D). The *qrt1-4*/*qrt1-4 gfat1-2/gfat1-2* plants completely stopped growing just after emergence of root radicle on GlcN-free media whereas *qrt1-4*/*qrt1-4* plants grew very well. Moreover, the growth rate of *qrt1-4*/*qrt1-4 gfat1-2/gfat1-2* plants increased in proportion to the GlcN concentration and reached the wild-type growth rate at 500 μM GlcN (GlcN-sufficient medium).

### *GFAT1 deficiency causes a decrease in protein* N*-glycation in Arabidopsis*

**Fig. 7.**
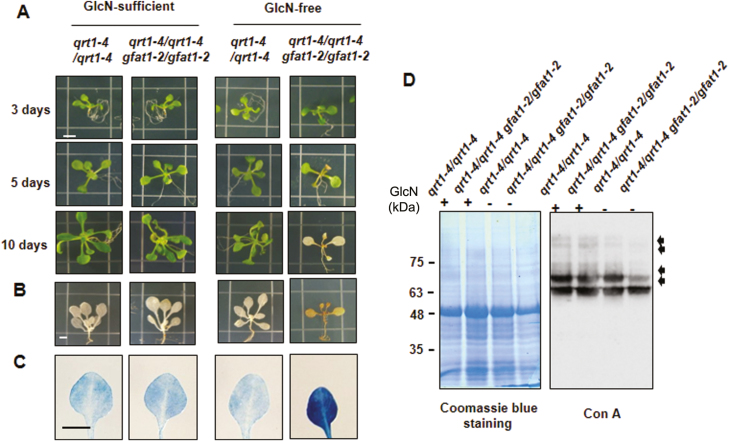
Symptoms of Arabidopsis plants due to GlcN deficiency. (A) Photographs of representative plants with the indicated genotype grown on GlcN-sufficient or -free medium. Seedlings were grown for 5 d on GlcN-sufficient medium (500 µM GlcN), followed by transfer to either GlcN-sufficient medium or GlcN-free medium, and then photographed 3, 5, and 10 d later. Five-day-old seedlings grown on GlcN-sufficient medium were transferred to either GlcN-sufficient medium or GlcN-free medium for 5 d and then stained with DAB (B) and trypan blue (C). (D) Reduction of protein *N*-glycosylation due to *AtGFAT1* deficiency. Five-day-old seedlings grown on GlcN-sufficient medium were transferred to GlcN-free medium for 3 d. Seedlings grown for the same period only on GlcN-sufficient medium were used as a control. Total proteins from of these seedlings were subjected to Con A blot analysis. The pattern of Coomassie Blue staining was used as a loading control. Compared with those in *qrt1-4*/*qrt1-4* plants, bands that displayed more weakly in *qrt1-4*/*qrt1-4 gfa1-2/gfa1-2* plants are indicated by arrows.

In order to examine the effect of GlcN deficiency on the growth of Arabidopsis, we transferred seedlings of *qrt1-4*/*qrt1-4* and *qrt1-4*/*qrt1-4 gfat1-2/gfat1-2* plants grown for 5 d in GlcN-sufficient medium into GlcN-free medium (Fig. 7A). Until the third day after the transfer, *qrt1-4*/*qrt1-4 gfat1-2*/*gfat1-2* plants displayed wild-type appearance not only on GlcN-sufficient medium but also on GlcN-free medium. Afterward, new leaves continued to emerge in *qrt1-4*/*qrt1-4* plants, whereas on the fifth day, no new leaves appeared and yellowing symptoms occurred in *qrt1-4*/*qrt1-4 gfat1-2/gfat1-2* plants. The *qrt1-4*/*qrt1-4 gfat1-2/gfat1-2* plants eventually turned brownish, leading to death at 10 d after the transfer to GlcN-free medium. To determine the possible causes of these symptoms, we stained *qrt1-4*/*qrt1-4* and *qrt1-4*/*qrt1-4 gfat1-2/gfat1-2* plants with DAB and trypan blue to detect the production of reactive oxygen species (ROS) and the cell death response, respectively (Fig. 7B, C). As expected, there was a significant increase in ROS and cell death at 5 d after the transfer to GlcN-free medium, but no difference was detected on GlcN-sufficient medium compared with those of wild-type plants. These results suggest that GlcN deficiency results in ROS production and cell death in Arabidopsis.

The final product of HBP, UDP-GlcNAc, is an essential substrate for supplying GlcN, the first two sugar moieties for protein *N*-glycosylation in the ER. Therefore, inhibition of the HBP flux is expected to have a significant effect on protein *N*-glycosylation in the ER (Nozaki *et al.*, 2012). To test this possibility, *qrt1-4*/*qrt1-4* and *qrt1-4*/*qrt1-4 gfat1-2/gfat1-2* mutant plants were grown for 5 d on GlcN-sufficient medium and then transferred to GlcN-free medium for 3 d. Total proteins from these plants were subjected to immunoblotting analysis using peroxidase-conjugated concanavalin A (ConA), a lectin with high affinity for oligomannose chains in protein *N*-glycans. Differences in Con A binding assay were detectable only between protein samples extracted from *qrt1-4*/*qrt1-4* and *qrt1-4*/*qrt1-4 gfat1-2/gfat1-2* mutant plants transferred to GlcN-free medium, with no difference between both plants grown on GlcN-sufficient medium (Fig. 7D). One of the strong bands for a protein of about 65 kDa showed little difference in intensity in *qrt1-4*/*qrt1-4 gfat1-2/gfat1-2* mutant plants, regardless of the presence of GlcN. However, band intensity for an ~70 kDa polypeptide was significantly decreased in *qrt1-4*/*qrt1-4 gfat1-2/gfat1-2* mutant plants transferred to GlcN-free medium, while it was found to remain unchanged on GlcN-sufficient medium. The additional decrease was observed in the band intensity of proteins with ~75 kDa and higher molecular mass in *qrt1-4*/*qrt1-4 gfat1-2/gfat1-2* mutant plants transferred to GlcN-free medium. These results strongly suggest that the reduction in protein *N*-glycosylation is at least partially involved in the aberrant phenotypes caused by *AtGFAT1* deficiency in Arabidopsis.

## Discussion

Under physiological conditions, 1–3% of intracellular glucose is metabolized through the HBP in eukaryotic cells (Buse, 2006). The first and rate-regulating step of the HBP is mediated by GFAT, transferring the amine moiety from glutamine to fructose-6-phosphate (Marshall *et al.*, 1991). Subsequently, GlcN-6-phosphate is processed in a four-step metabolic pathway, leading to the production of UDP-GlcNAc, the final product that serves as an essential amino sugar donor for glycosylation of lipids and proteins. It also provides GlcNAc residues for structural polymers such as chitin in the fungal cell wall and the exoskeleton of arthropods (Kato *et al.*, 2002). Despite many studies on genes encoding other enzymes involved in the HBP in plants, no studies have been reported to date on the *GFAT* gene in plants.

T-DNA insertions in the exon of *AtGFAT1* had a lethal effect (Table 1; Fig. 2A), which is consistent with previous reports showing the lethal phenotype of Arabidopsis mutants lacking glucosamine-6-phosphate *N*-acetyltransferase (GNA) and *N*-acetylglucosamine-1-P uridylyltransferase (GlcNAc1pUT) activity catalysing the second and last step of HBP, respectively (Riegler *et al.*, 2012; Chen *et al.*, 2014). However, T-DNA insertion in *OsGNA1* of rice caused a defect in root elongation, but not a lethal phenotype (Jiang *et al.*, 2005). The roots of the *osgna1* rice mutant were revealed to contain about 10% of the UDP-GlcNAc content of wild-type rice, which is likely to enable the *OsGNA1* rice mutant to survive.

In this report, we show that *AtGFAT1* deficiency causes abnormal phenotypes in almost every developmental process in Arabidopsis and that most of them were rescued by exogenous GlcN supplementation. For example, T-DNA insertion into the exon of *AtGFAT1* completely inhibited germination of pollen grains as well as polar deposition of callose and pectin in pollen grains (Fig. 2B, C, D). The exogenous GlcN supplement also rescued germination of pollen grains bearing the *gfat1-2* allele (Fig. 6A). Although *qrt1-4*/*qrt1-4 gfat1-2/gfat1-2* plants grown on GlcN-free medium ceased further growth after root radicle emergence from the seed coat, no detectable defects were observed on a GlcN-sufficient medium (Fig. 6C). The *ATGFAT1* deficiency had little effect on protein *N*-glycosylation when GlcN was supplemented, while it was significantly impaired in *gfat1-2* homozygous mutant plants transferred to GlcN-free medium (Fig. 7D). Taken together, these results strongly suggest that most of the defects of *AtGFAT1* mutations are attributed to blocking the biosynthesis of GlcN in Arabidopsis.

Several recent studies have addressed how the interaction between the HBP and the UPR was modulated through regulation of the *GFAT* gene, encoding the first and rate-regulating enzyme in animal systems. For example, Denzel *et al.* (2014) demonstrated that GOF mutation in *gfat-1* resulted in elevated UDP-GlcNAc contents in *C. elegans*, leading to Tm resistance and longevity. The HBP flux was also activated in mice by Xbp1s, a transcription factor, which is induced by the UPR, leading to attenuation of I/R injury of heart (Wang *et al.*, 2014). They also showed that an HBP intermediate supplement itself significantly reduced cell death by I/R injury. Moreover, I/R injury was shown to induce the UPR in mice (Llorente *et al.*, 2013). Consistent with these previous reports in the animal system, *AtGFAT1* expression was also significantly induced by Tm-mediated ER stress (Figs 1C, 3A). As shown in nematode and mouse, overexpression of *AtGFAT1* in Arabidopsis produced about twice as much GlcN and UDP-GlcNAc as the wild-type under normal conditions (Fig. 3B, C). In addition to *AtGFAT1* overexpression, an exogenous GlcN supplement also conferred Tm resistance to plants (Fig. 4A), as in *C. elegans*.

However, *AtGFAT1* overexpression in Arabidopsis was different from that in *C. elegans* in that it had little effect on every developmental process including longevity under normal growth conditions (Fig. 5). *gfat-1* GOF mutation in *C. elegans* was revealed to enhance ERAD protein expression and autophagic activity (Denzel *et al.*, 2014). In mice, Xbp1s stimulation of HBP activity was revealed to increase in *O*-GlcNAc protein modification (Wang *et al.*, 2014). Mutation in the *EMeg32* gene encoding GNA in mice cells displayed decreased UDP-GlcNAc levels and apparent lack of *O*-GlcNAc modification of cytosolic proteins (Boehmelt *et al.*, 2000). These reports suggest that activation of HBP flux by ER stress is conserved in many species including nematode, mammals, and plants. However, the elevated levels of UDP-GlcNAc appear to bring different biological effects through particular glycosylation of a distinct set of proteins depending on each species.

Conceivably, ROS production and cell death due to *AtGFAT1* deficiency would be relevant to various defects in *gfat1* mutant and transgenic plants. A decrease in protein *N*-glycosylation by *AtGFAT1* deficiency would be also one of the major causes of aberrant phenotypes in *gfat1* mutant and transgenic plants. However, given the inhibition of pollen germination and pollen tube growth by mutations of *SETH1* and *SETH2*, encoding the subunits of the GPI–GnT complex (Lalanne *et al.*, 2004), we cannot rule out the possibility that *AtGFAT1* deficiency also impairs other types of post-translational modifications of proteins in *gfat1-2*/*gfat1-2* plants. Therefore, it will be interesting to identify endogenous proteins that display significant changes in protein glycosylations in *gfat1-2*/*gfat1-2* plants and to address how these alterations affect protein targeting, stability, and activity.

## Supplementary data

Supplementary data are available at *JXB* online.

Fig. S1. Multiple alignment of amino acid sequences of L-glutamine D-fructose-6-phosphate amidotransferase (GFAT).

Fig. S2. The *gaft1-2* mutation has no effect on morphology, nuclear division, and viability of pollen quartets at the tricellular stage.

Fig. S3. Complementation of male gametophytic defect of *gfat1-2*/+ plants by introduction of *pGFAT1::GFAT1*.

Fig. S4. Construction of *AtGFAT1* overexpression and RNAi suppression lines.

Fig. S5. Schematic representation of predicted promoter sequence of *AtGFAT1*.

Fig. S6. The effect of DTT on the primary root growth of wild-type, *gfat1-1*, *AtGFAT1* OE, and RNAi lines.

Table S1. List of primers used in this work.

Table S2. Number of siliques per plant and seeds per silique in wild-type, *AtGFAT1* OE, and RNAi lines.

Supplementary Data

## Abbreviations

GFAT: glutamine:fructose-6-phosphate amidotransferase
GlcN: glucosamine
GlcN-6-P: glucosamine-6-phosphate
GlcNAc: *N*-acetylglucosamine
GNA: glucosamine-6-phosphate *N*-acetyltransferase
GnT: *N*-acetylglucosaminyltransferase
GOF: gain of function
GPI: glycosylphosphatidylinositol
HBP: hexosamine biosynthetic pathway
I/R: ischemia–reperfusion
OGT: *O*-GlcNAc trbsferase
UDP-GlcNAc: UDP-*N*-acetylglucosamine
UPR: unfolded protein response
Xbp1s: spliced X-box binding protein 1
