# Supplementary material for: Deficiency of AtGFAT1 activity impairs growth, pollen germination and tolerance to tunicamycin in Arabidopsis
Source: J Exp Bot. 2019 Feb 18;70(6):1775–87. doi: 10.1093/jxb/erz055 (PMC6436160; doi:10.1093/jxb/erz055)
Supplement: Supplementary Data [file erz055_suppl_supplementary_data.pdf]

I'm sorry that I can't make it clear. The given and family names for the authors are highlighted in blue and yellow, respectively, as shown below, and the middle name is not highlighted.

Kien Van **Vu,**

Chan-Young Jeong,

Thuy Thi **Nguyen,**

Trang Thi Huyen **Dinh,**

Hojoung Lee,

Suk-Whan Hong

I would appreciate it if the name of the funding organization in the Acknowledgements remains unchanged.

Table S1. List of primers used in this work

Name	Oligo sequence 5′ to 3′	Restriction enzyme site	Purpose
LBb1	GCGTGGACCGCTTGCTGCAACT		Left border primer for identification of T-DNA
P1 (GFAT1-1:F)	TAGGGACAGTGGCTAGTGGCC		Genotyping
P2 (GFAT1-1:R)	GCTCACCATGAGTAGCCCACC		
P3 (GFAT1-2:F)	CTCAATCTTCTGGTCCTGGAG		
P4 (GFAT1-2:R)	ACCGCAGTTGCTTACAAACCC		
P5 (GFAT1-3:F)	GCGTGGATAGTGGCACAGTTG		
P6 (GFAT1-3:R)	TCAGCCCACCTAAGAGGACGG		
QRT1-4:F	GAGCTGTTGATATGGTTCCTG		
QRT1-4:R	CTAAGTAAATCTGCCCAGTAC		
pGFAT1:F	CAATGAAGCTTTACTATTCTG	HindIII	Making *pGFAT1::GUS* and *pGFAT1::GFAT1* constructs
pGFAT1:R	CGGATCCTTGAAAGAAAATGTTTCTCAG	BamHI	
GFAT1 cDNA:F	CGGATCCATGTGTGGAATCTTCGCG	BamHI	Making *pGFAT1::GFAT1* and *GFAT1* OE constructs
GFAT1 cDNA:R	CGAGCTCCTATTGAGTAGTCACACT	SacI	
GFAT RNAi Sense:F	GGGCGCGCCGCTACTCATGGTGAGCCAGCT	AscI	Making *GFAT1* RNAi construct
GFAT RNAi Sense:R	GATTTAAATGTTCCACTGAAGCAGGTCTCG	SwaI	
GFAT RNAi antisense:F	GTCTAGAGCTACTCATGGTGAGCCAGCT	XbaI	
GFAT RNAi antisense:R	GGGATCCGTTCCACTGAAGCAGGTCTCG	BamHI	
ACTIN2:F	GTGTGTCTTGTCTTACTCGGTTCG		RT-PCR and qRT-PCR
ACTIN2:R	AATAGCTGCATTGTCACCCGATACT		
GFAT1:F	ACAGTGAAGGAATACTTGCAG		
GFAT1:R	TTGAGTAGTCACACTCTTTGC		
CNX1:F	CTCGTCGCCATTGTGGTTGTA		
CNX1:R	CCGTCTTCACATACATATTCC		
BIP1/2:F	AATGCCCTGGAGACATACGTG		
BIP1/2:R	CTCCTCAGTCGATGATTCTCC		
BIP3:F	GAAGATGACGAGGAACAAAGC		
BIP3:R	TTCCCAAGAAAGCTTCAGCTG		

Table S2. Number of siliques per plant and seeds per silique in wild-type, *gfat1-1*, *AtGFAT1* overexpression and RNAi lines

Lines	Number of seeds per one silique	Number of siliques per one plant
Col-0	50.2 ± 2.1	108.6 ± 12.3
gfat1-1	51.5 ± 1.7	106.2 ± 9.5
GFAT1 OE	50.0 ± 1.6	104.7 ± 11.6
GFAT1 RNAi	29.8 ± 2.2	29.5 ± 4.9

The values are mean ± SD (n = 50 for seeds per one silique; n = 10 for siliques per one plant)

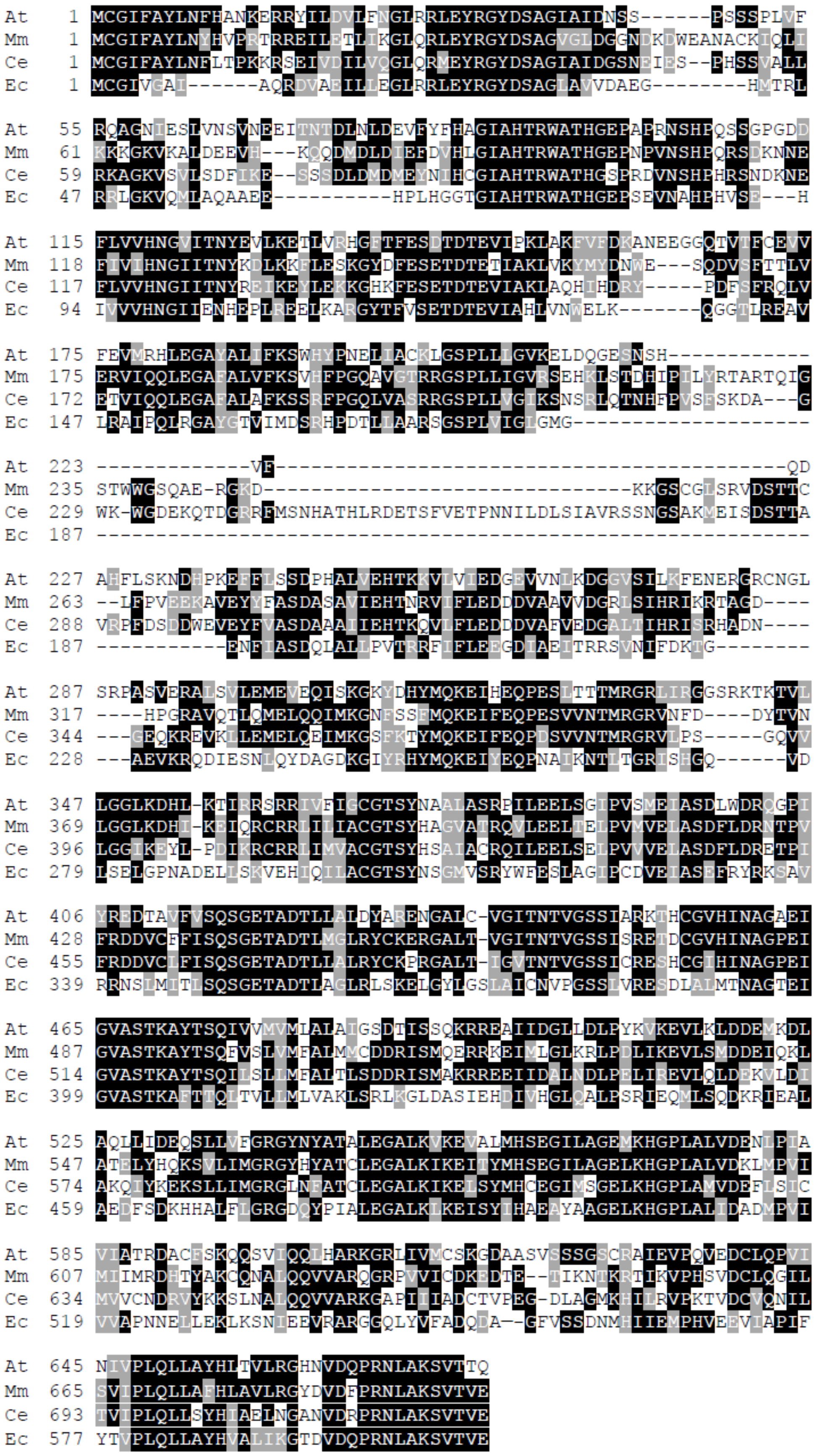

Supplementary Figure S1. Multiple alignment of amino acid sequences of L-glutamine D-fructose-6-phosphate amidotransferase (GFAT). At, *Arabidopsis thaliana* GFAT (At3g24090); Mm, *Mus musculus* GFAT (P47856); Ce, *Caenorhabditis elegans* GFAT (Q19130); Ec, *Escherichia coli glmS* GFAT (P17169). The black-filled boxes and the gray-filled boxes indicate identical and similar residues, respectively.

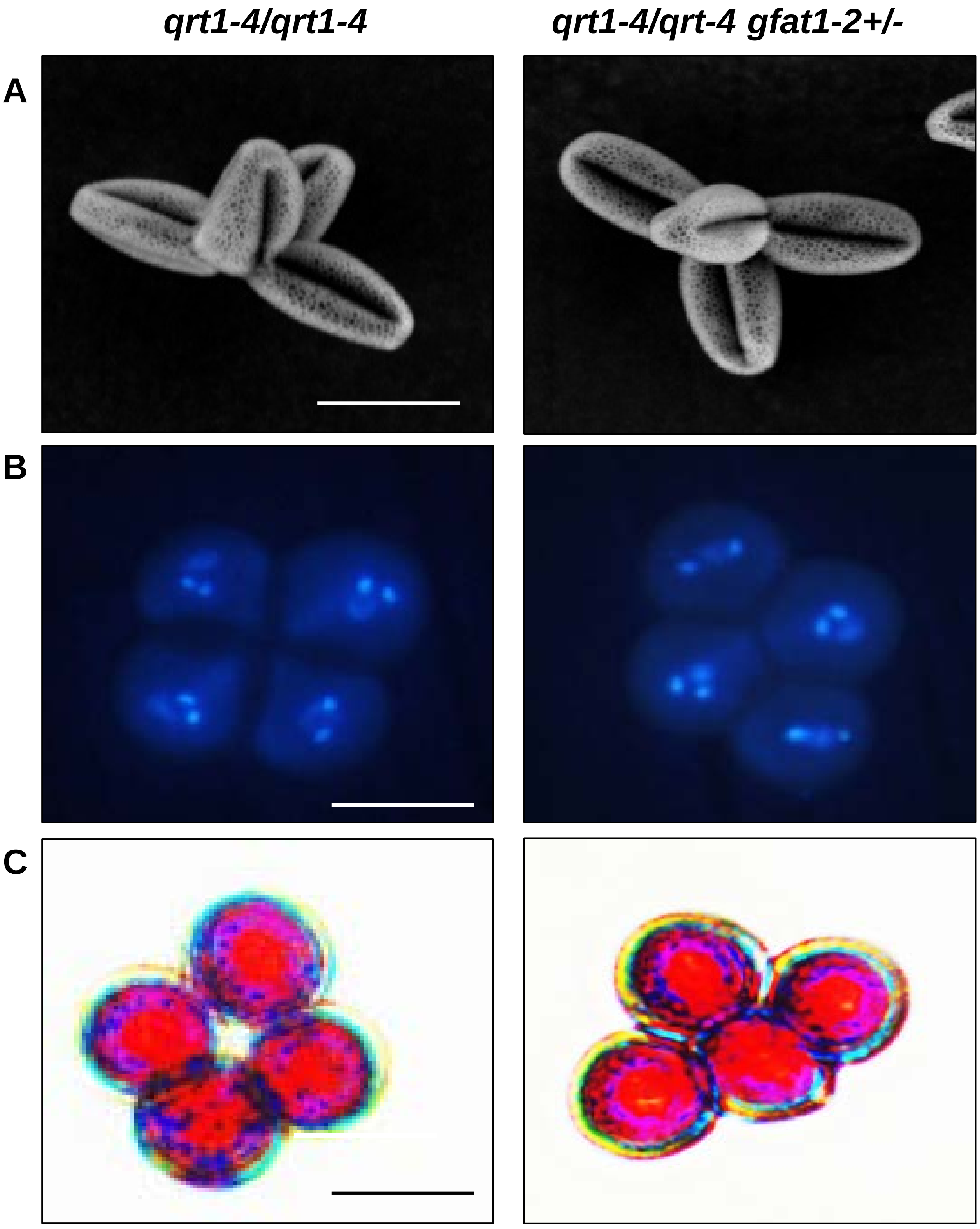

Supplementary Figure S2. The *gaft1-2* mutation has no effect on morphology, nuclear division, and viability of pollen quartets at the tricellular stage. (A) Scanning electron microscopy (SEM) imaging of pollen quartets from *qrt1-4*/*qrt1-4* and *qrt1-4*/*qrt1-4 gfat1-2*/+ plants. (B) DAPI staining analysis of pollen quartets. (C) Alexander staining analysis of pollen quartets. Scale bars: 20 µm.

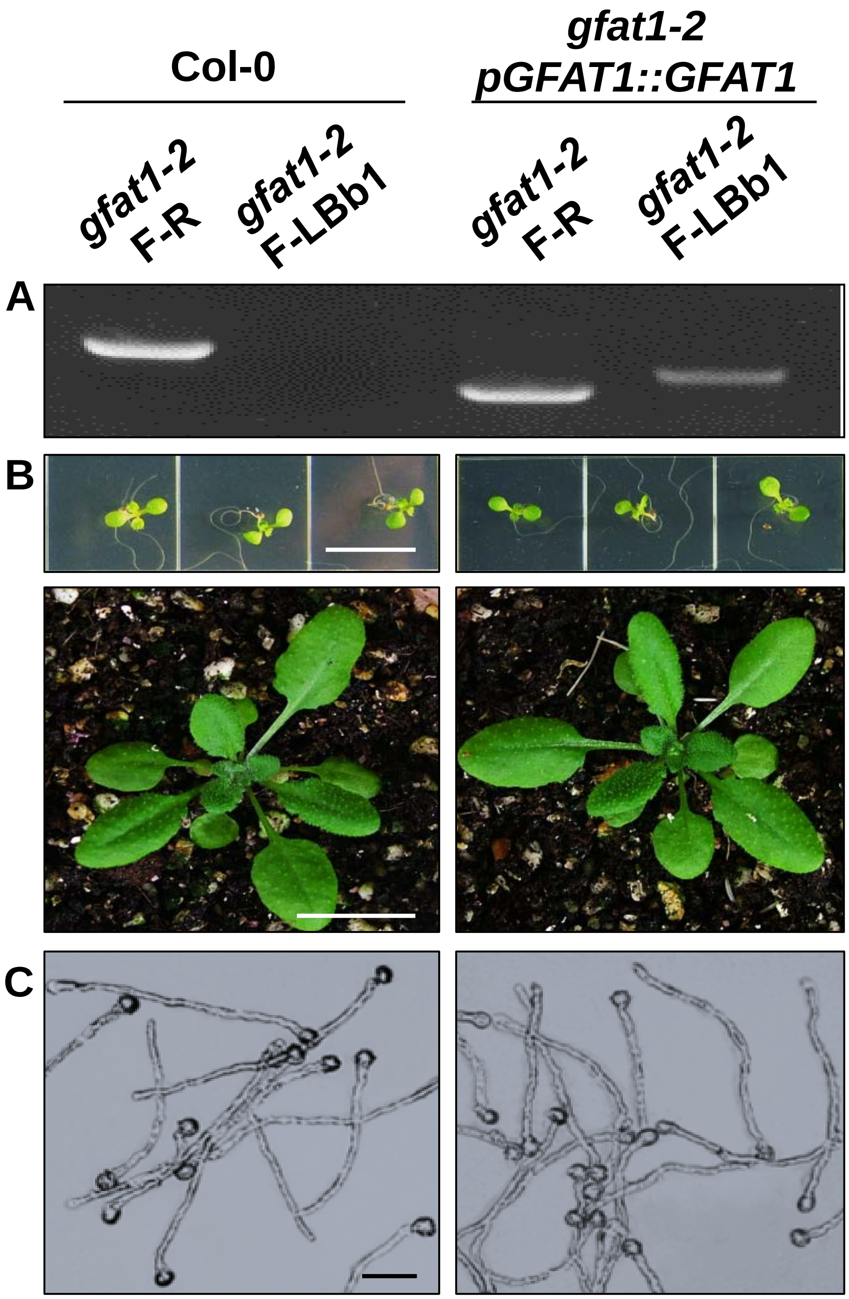

Supplementary Figure S3. Complementation of male gametophytic defect of *gfat1-2*/+ plants by introduction of *pGFAT1::GFAT1*. (A) PCR analysis for distinguishment of *AtGFAT1* cDNA and genomic DNA using the *gfat1-2* F and *gfat1-2 R* primers and for identification of T-DNA insertion using the *gfat1-2* F and LBb1 primers. Genomic DNA was extracted from leaves of 4-week-old plants. As expected, the 641-bp T-DNA specific and the 475-bp *AtGFAT1* cDNA-specific PCR products were amplified from *gfat1-2/gfat1-2* plants. However, the 915-bp gene-specific PCR products, including intron, was not amplified in the *gfat1-2 /gfat1-2* mutant plants. In contrast, wild-type plants yielded only the 915-bp gene-specific PCR products including intron. (B) 10-day-old and 4-week-old plants of the complementation line (*gfat1-2 pGFAT1::GFAT1*) displayed no detectable differences under normal conditions compared to the wild-type plant (Col-O). (C) *In vitro* germination of pollen grains from wild-type and the complementation plants. Scale bars: 1.5 cm (B), 50 μm (C).

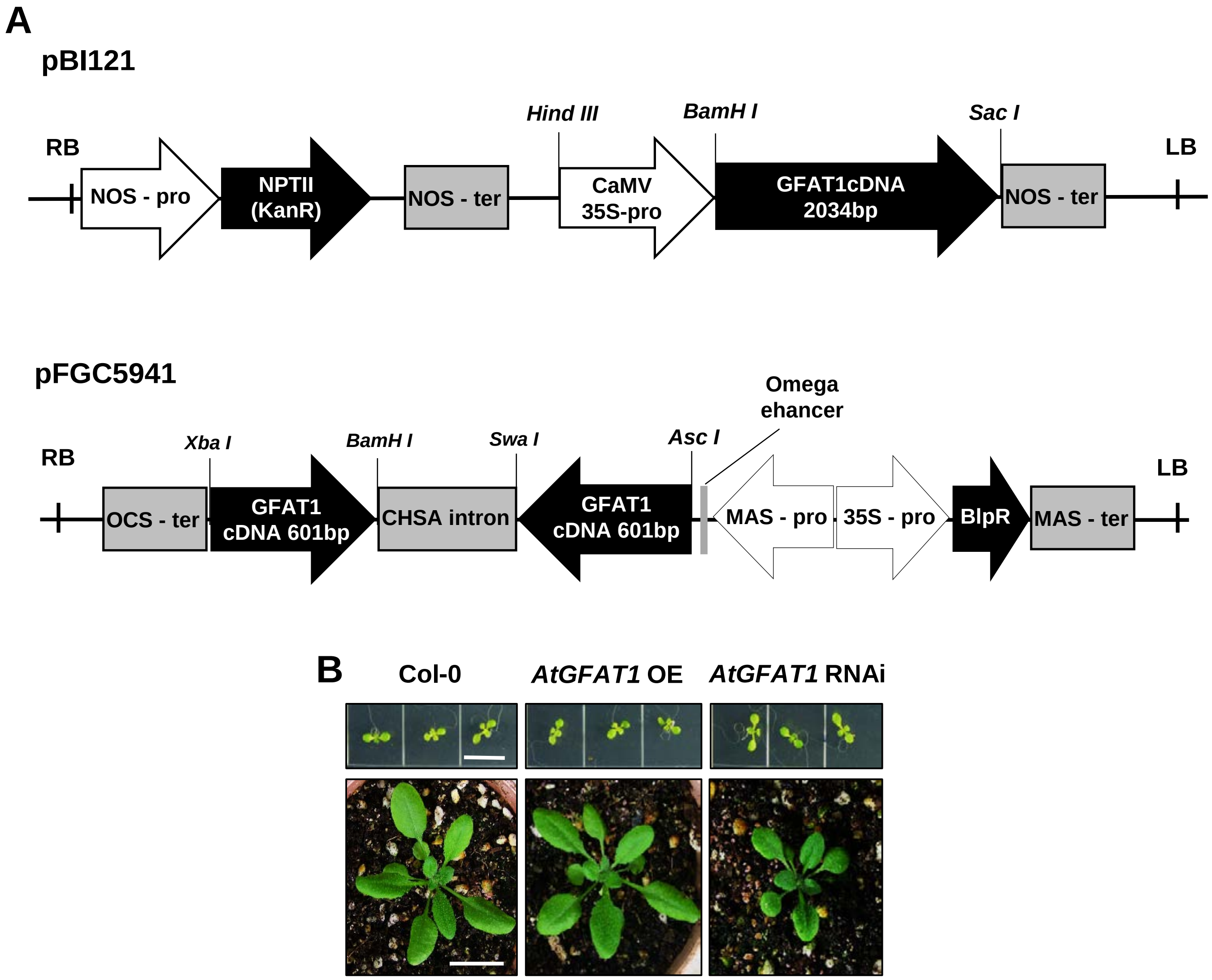

Supplementary Figure S4. Construction of *AtGFAT1* overexpression and RNAi suppression lines. (A) The schematic diagrams for construction of *AtGFAT1* overexpression and *RNAi* constructs. Full-length *AtGFAT1* cDNA was placed under the control of the cauliflower mosaic virus 35S (CaMV35S) promoter in pBI121 vector to make the *AtGFAT* overexpression (OE) construct, and the *AtGFAT1* RNA interference (RNAi) construct was produced containing an inverted repeat of a 601 bp DNA fragment of *AtGFAT1* cDNA in the pFGC5941 vector. These constructs were transformed into wild-type *Arabidopsis* using the Agrobacterium strain GV3101. We selected three independent *h*omozygous lines of transgenic plants carrying a single copy of the transformed construct at T3 generation for detailed analyses. (B) Photographs of representative 10-day-old and 4-week-old plants of wild-type and transgenic plants. Scale bars: 1.5 cm.

GFAT1 promoter

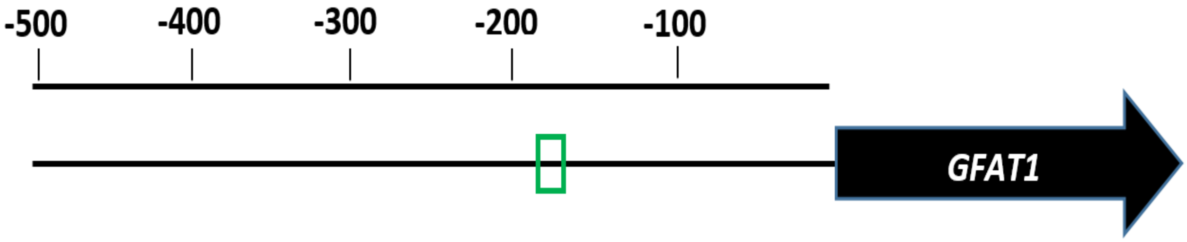


```
-350   aaaacagagagattcctaattatgaattgatttttactgtatttaattat
-300   gaattgatctgaacttagggacagtggctagtggcctatatcagcacgtg
-250   acactagccacgcgtagcggttccgctccaacaaaaagcaaactcgcta
-200   tctgtccaatcaggaacaaccacgtcatagctcctgacagaagtgaaaaa
-150   gacgaaactatcCaaacttatggatcgtatttacaaagtcaaacccaaat
-100   ctgagtcaaaaaataagaatctcaccgttccttacttcttcttcatcttc
-50    cctcgaaaaactgagaaacatttctttcaaaagagagattacaacaacc
+1     ATG
```

Supplementary Figure S5. Schematic representation of predicted promoter sequence of *AtGFAT1*. Line and arrow indicate the promoter and the coding region of *AtGFAT1*, respectively. Green box indicates the relative location of the ERSE motif in the *AtGFAT1* promoter. The nucleotide sequences of the ERSE motif in the promoter are represented in green color. The A of ATG, the translation initiation codon, is set to +1 to determine the position of the promoter sequence.

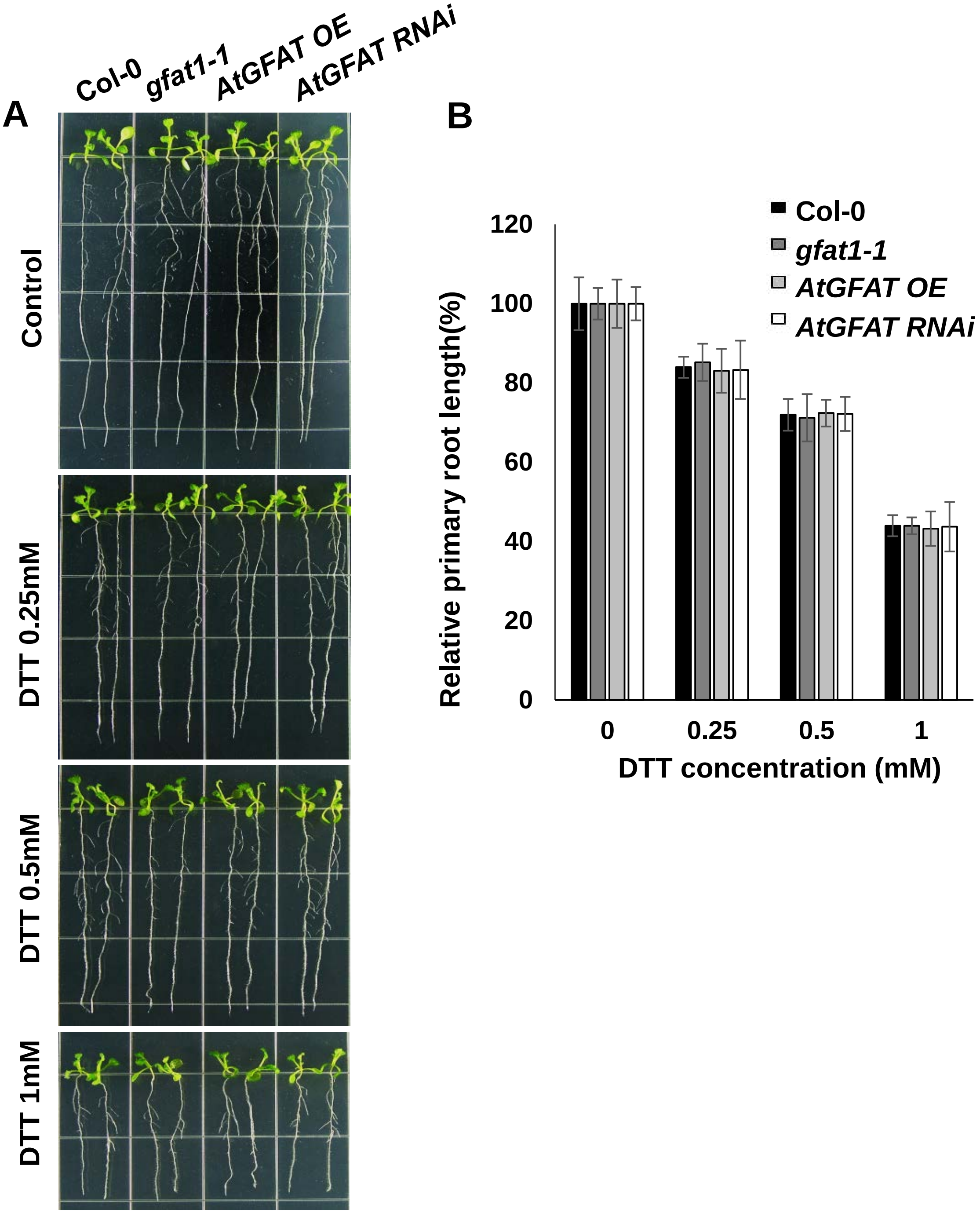

Supplementary Figure S6. The effect of DTT on the primary root growth of wild-type, *gfat1-1*, *AtGFAT1* OE, and RNAi lines. (A) Wild-type, *gfat1-1*, *AtGFAT1* OE, and RNAi seedlings were vertically grown for 10 days on ½ MS media supplemented with a range of DTT concentrations. Representative pictures of 10-day-old seedlings were taken. (B) The primary root length grown under DTT treatment was represented as a relative value for the root grown in normal conditions. Values represent means ± SE of three repeats ($n \geq 20$ per repeat).
